# Live-cell STED nanoscopy of mitochondrial cristae

**DOI:** 10.1038/s41598-019-48838-2

**Published:** 2019-08-27

**Authors:** Till Stephan, Axel Roesch, Dietmar Riedel, Stefan Jakobs

**Affiliations:** 1 Department of NanoBiophotonics, Max Planck Institute for Biophysical Chemistry, 37077 Göttingen, Germany; 2 Laboratory of Electron Microscopy, Max Planck Institute for Biophysical Chemistry, 37077 Göttingen, Germany; 3 Clinic of Neurology, University Medical Center Göttingen, 37075 Göttingen, Germany

**Keywords:** Fluorescence imaging, Mitochondria, Super-resolution microscopy

## Abstract

Mitochondria are highly dynamic organelles that exhibit a complex inner architecture. They exhibit a smooth outer membrane and a highly convoluted inner membrane that forms invaginations called cristae. Imaging cristae in living cells poses a formidable challenge for super-resolution light microscopy. Relying on a cell line stably expressing the mitochondrial protein COX8A fused to the SNAP-tag and using STED (*st*imulated *e*mission *d*epletion) nanoscopy, we demonstrate the visualization of cristae dynamics in cultivated human cells. We show that in human HeLa cells lamellar cristae are often arranged in groups separated by voids that are generally occupied by mitochondrial nucleoids.

## Introduction

Mitochondria form tubular and highly dynamic networks in mammalian cells that constantly undergo fusion and fission events^1,2^. They are double-membrane organelles that exhibit a smooth outer membrane and a highly convoluted inner membrane. Cristae are invaginations of the inner membrane that generally adopt tubular or lamellar shapes and project into the matrix space. The cristae architecture adapts to different cellular conditions and is changed upon various stimuli^3,4^. However, the actual cristae dynamics are poorly understood. Electron microscopy (EM) routinely provides the required resolution to visualize cristae, but it is restricted to fixed samples.

The study of cristae in living cells requires light microscopy; a challenge for studying cristae dynamics is the small size of the mitochondria. The diameter of mitochondrial tubules is generally between 200 and 700 nm and, in many mammalian cell types, the crista-to-crista distance can be below 100 nm. Hence, because of the diffraction limit in optical microscopy (~250 nm, depending on the wavelength), visualization of individual cristae by light microscopy has been a notorious challenge^5^. The development of various forms of super-resolution microscopy that enable a substantial better or even diffraction-unlimited (nanoscopy) resolution paved the way to overcome this problem. For a detailed discussion of the various techniques see expert reviews^6–8^.

In fixed cells, cristae were first visualized by light microscopy with isoSTED nanoscopy^9^. Using structured illumination microscopy (SIM) providing a resolution of around 100 nm, mitochondrial sub-structures were resolved in living cells^10–12^. However, the discrimination between individual cristae and groups of cristae in human mitochondria remained difficult because of the limited resolution.

Diffraction-unlimited nanoscopy provides a resolution of substantially better than 100 nm^7^. So far, the overall mitochondrial dynamics and the cristae movements, difficulties in labelling and concerns on light induced photo-toxicity have hampered the visualization of single cristae dynamics using live-cell nanoscopy^7,13,14^.

In this study we overcome this problem by using diffraction-unlimited STED super-resolution microscopy (STED nanoscopy) in combination with a genome edited human cell line that enables labeling of the cristae with a silicon rhodamine dye. Thereby, we unequivocally visualize the dynamics of individual cristae in human cells using nanoscopy.

## Results

### Visualization of the cristae architecture in living cells

We have generated a human HeLa cell line that stably expresses the full length cytochrome *c* oxidase subunit 8A (COX8A), an integral protein of the mitochondrial inner membrane, fused to the SNAP-tag^15^. The SNAP-tag is a self-labeling protein that directly reacts with a labeling compound such as a cell permeable fluorophore that is added to the growth medium^16^. Thereby the SNAP-tag provides flexibility in the choice of the fluorophore. As COX8A is a subunit of complex IV of the respiratory chain, it is expected to be preferentially localized in the crista membrane^17,18^. Fusions to COX8A have been used in several studies to highlight mitochondria, without affecting mitochondrial function^19^. The fusion gene was targeted to the chromosomal AAVS1 (*a*deno-*a*ssociated *v*irus integration *s*ite 1) Safe Harbor Locus using a CRISPR/Cas9 based genome editing strategy, ensuring largely constant expression levels^20,21^. By using this fusion construct we target the SNAP-tag to the mitochondrial inner membrane.

**Figure 1:**
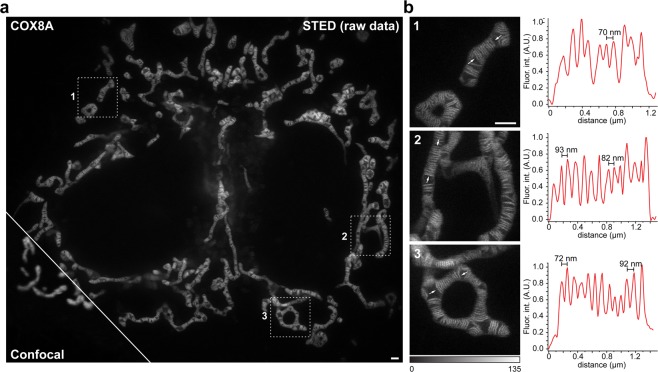
Live-cell Stimulated Emission Depletion (STED) nanoscopy of mitochondrial cristae in HeLa cells. HeLa cells stably expressing COX8A-SNAP fusion proteins were labeled using SNAP-Cell SiR and visualized with STED nanoscopy. (**a**) Overview of HeLa cells. Shown is a comparison between confocal and STED resolution. (**b**) Left: Magnifications of the areas indicated in the overview image. Right: Fluorescence intensity line profiles measured as indicated in the magnifications. STED images show unprocessed raw data without background subtraction. Scale bars: 1 µm.

**Figure 2:**
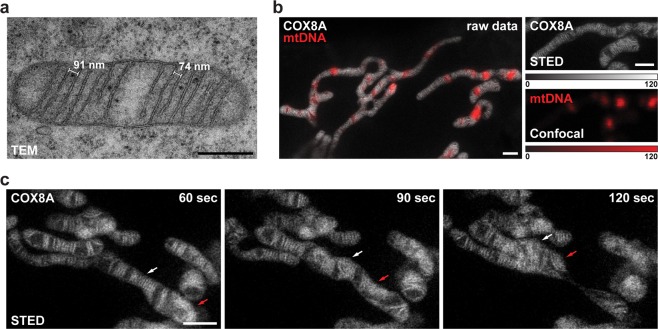
Dynamics of mitochondrial cristae. (**a**) Transmission electron microscopy (TEM) of mitochondria from HeLa cells. (**b**) Dual-color live-cell imaging of mitochondria. HeLa cells stably expressing COX8A-SNAP fusion proteins were labeled using SNAP-Cell SiR. In addition, mtDNA was labeled with PicoGreen (red). The cristae were visualized by STED nanoscopy, the nucleoids by confocal scanning light microscopy. (**c**) Live-cell time-lapse STED nanoscopy of mitochondria. Mitochondria were recorded every 15 seconds. Shown are three frames out of ten. Arrows indicate the behavior of two separated groups of cristae during the fission of a mitochondrion. The whole image series is shown in Supplementary Movie S1. (b) Shows unprocessed raw data without background subtraction; in (c) photobleaching was compensated by adapting the color table. Scale bars: a: 0.5 µm; b,c: 1 µm.

Labeling of living cells by adding the cell-permeant dye SNAP-Cell SiR to the medium^22^ resulted in brightly fluorescent mitochondria (Fig. 1a; Supplementary Fig. S1). As expected, diffraction-limited confocal recordings did not reveal any sub-mitochondrial structures, whereas with STED nanoscopy (excitation: 640 nm, STED: 775 nm), we were able to resolve individual cristae in mitochondria of living HeLa cells with ~50 nm resolution (Fig. 1). We measured the crista-to-crista distances in 15 mitochondria of different living cells, and found a distance of 70 to 100 nm between closely spaced cristae (Fig. 1b, Supplementary Fig. S2a). Within groups of cristae, the mean crista-to-crista distance was 123 nm (Supplementary Fig. S2a,c), which is fully in line with the mean crista-to-crista distance of 120 nm measured on electron micrographs recorded from the same cell line (Fig. 2a, Supplementary Fig. S2b,c). Using live-cell STED nanoscopy, we were able to record entire cells, thereby getting an overview on the cristae architecture of mitochondria with different shapes or with different subcellular positions (Fig. 1).

### Mitochondrial nucleoids occupy the voids between groups of cristae

The cristae frequently occurred in groups, separated by voids of several hundred nanometer size. Mitochondrial DNA (mtDNA) is compacted into nucleoprotein complexes termed mitochondrial nucleoids. We used the cell-permeant dye PicoGreen to label the mtDNA and analyzed the location of the mitochondrial nucleoids with respect to the cristae in live mitochondria. Surprisingly, we found that in this cell line most free spaces in the mitochondrial matrix that were devoid of cristae were occupied by mitochondrial nucleoids (Fig. 2b; Supplementary Fig. S3a,b). This finding was fully corroborated by dual-color STED images of chemically fixed HeLa cells labeled with antisera against ATP5I, a subunit of the F_1_F_o_-ATP synthase, and against double stranded DNA (Supplementary Fig. S3c).

### Time lapse imaging of cristae dynamics

Our labeling strategy allowed us to capture individual cristae and their dynamics across larger fields of view. By this we could, for example, capture the dynamics of cristae apparently moving in groups during the fission of a mitochondrial tubule (Fig. 2c; Supplementary Movie S1). Due to the flexibility of the beam-scanning STED imaging scheme, the size of the recorded field of view can be adapted, facilitating video sequences with frame rates in the second range. Even at this temporal resolution, we observed an unexpected level of cristae movements (Supplementary Movies S2 and S3). When the cells were additionally labeled with the live-cell DNA stain PicoGreen, we could additionally follow the movements of the nucleoids within the mitochondria (Supplementary Movie S4). Due to photobleaching (Supplementary Fig. S3d), the imaging sequences were typically limited to 10–20 frames. We note that after 20 frames the mitochondria generally did not show signs of photostress, which is in line with the observation that STED nanoscopy is live-cell compatible^14^. We envisage that with the development of new dyes that are less prone to photobleaching under live-cell conditions, much longer image sequences should be possible.

## Discussion

The intricate mitochondrial membrane architecture is vital for the functioning of mitochondria as cellular powerhouses. It is widely accepted that the mitochondrial cristae are dynamic structures that are remodeled upon various cellular stimuli, but also upon apoptosis and during ageing^4,23–25^. The mechanisms of mitochondrial cristae biogenesis are debated and different mechanisms for this process have been suggested^26–28^. Currently, very little is known about the actual dynamics of mitochondrial cristae in these processes since a clear visualization of the cristae structure in living cells has been a notorious challenge in the past.

To better analyze the formation of cristae and their structure and dynamics under different cellular conditions, a new approach for high-resolution live-cell imaging of mitochondrial cristae is urgently needed. We suggest that the COX8A-SNAP cell line in combination with nanoscopy will be a valuable resource to study cristae dynamics and cristae biogenesis. Our dual-color recordings of cristae and mitochondrial nucleoids revealed that in the cell line we used, nucleoids generally occupy voids between groups of cristae. Mitochondrial nucleoids, because of their low contrast, are usually invisible in conventional EM, although experimentally advanced methods such as electron cryo-tomography^29^ or correlative light-electron microscopy^30^ allowed to visualize nucleoids next to cristae in fixed cells.

We note that our images have been recorded with a commercial STED microscope, which will allow also non-specialized laboratories to record cristae dynamics. STED microscopy might be particularly suitable for the study of cristae dynamics as its scanning approach allows the fast imaging of small fields of view with the possibility to adjust the required optical resolution. When focusing only on a single mitochondrion, the photostress is minimal since the rest of the cell is not illuminated. In this study, we used a STED microscope that improved the resolution in the lateral plane, but was diffraction limited along the z-axis (2D STED nanoscopy). As the HeLa cell line used primarily exhibits lamellar cristae that are oriented perpendicular to the growth surface, this imaging mode was well suited. Other cell types may exhibit tubular or more complex cristae arrangements^3,18,28,31^ and therefore will require 3D nanoscopy in order to provide a full view on the orientation of the cristae in the mitochondria. Clearly, also other nanoscopies including RESOLFT/non-linear SIM^32–34^ are likely to be able to visualize individual cristae dynamics. As diffraction-limited linear SIM, enabling a resolution of 100 nm, has been previously used to visualize cristae or groups of cristae in living cells^10–12^, it will be interesting to compare the different imaging modalities on the same cell type.

While this paper was in review, another study reported on the development of the fluorescent probe MitoPB Yellow, which labels mitochondrial cristae and facilitates their visualization using STED nanoscopy^35^. As MitoPB Yellow binds to membranes, its use does not rely on the expression of fusion proteins, but provides no freedom in the choice of the wavelengths used for imaging. The SNAP-tag that we used to tag COX8A allows to change the fluorophore according to the requirements of the different light microscopy techniques. This versatility will facilitate an immediate comparison of different fluorophores, labelling strategies and imaging modalities on a dynamic and challenging live-cell sample. Moreover, the chemical fixation of the double membraned mitochondria is often difficult and we expect this cell line will be a valuable resource for evaluating and systematically improving fixation conditions for optical microscopy.

## Material and Methods

### Cloning of plasmids

To generate the donor plasmid AAVS1-Blasticidin-CAG-COX8A-SNAP, the plasmid AAVS1-Basticidin-CAG-Flpe-ERT2 was first linearized by using the restriction endonucleases SalI and EcoRV. AAVS1-Blasticidin-CAG-Flpe-ERT2 was a gift from Su-Chun Zhang (Addgene plasmid #68461; http://n2t.net/addgene:68461; RRID:Addgene_68461).

COX8A-SNAP was amplified by PCR from pSNAPf-COX8A (New England Biolabs, Ipswich, MA, USA) using the primers given below and subsequently integrated into the linearized plasmid by Gibson assembly.

Primers for donor Plasmid:

COX8A-fwd: TCTCATCATTTTGGCAAAGAATTCGTCGACGCCGCCACCATGTCCGTCCTGACGCCG

COX8A-rev: GAGGTTGATTATCGATAAGCTTGATATCTTAATTAACCTCGAGTTTAAACGCGGATC

The gRNA plasmid PX458-AAVS1 was derived from PX458. pSpCas9(BB)-2A-GFP (PX458) was a gift from Feng Zhang (Addgene plasmid #48138; http://n2t.net/addgene:48138; RRID:Addgene_48138). In brief, oligonucleotides were annealed by primer annealing and integrated into PX458 after linearization with the *Bbs*I restriction endonuclease.

Oligonucleotides for gRNA plasmid:

AAVS1-gRNA-fw: CACCGTGTCCCTAGTGGCCCCACTG

AAVS1-gRNA-rev: AACCAGTGGGGCCACTAGGGACAC

### Cell culture

HeLa cells were cultured in DMEM containing 4.5 g/L glucose and GlutaMAX™ additive (Thermo Fisher Scientific) supplemented with 100 U/mL penicillin and 100 µg/mL streptomycin (Merck Millipore, Burlington, MA, USA), 1 mM sodium pyruvate (Sigma Aldrich) and 10% (v/v) fetal bovine serum (Merck Millipore).

### Generation of a stable cell line

To generate the stable cell line, HeLa cells were co-transfected with the plasmids AAVS1-Blasticidin-CAG-COX8A-SNAP and PX458-AAVS1 using the jetPRIME transfection reagent (Polyplus, Illkirch, France). Starting two days after transfection, the cells were selected using DMEM containing 10 µg/mL Blasticidin S (Invivogen, Toulouse, France) for 7 days. Two weeks after transfection, the cells were stained with DMEM containing 1 µM SNAP-Cell SiR (New England Biolabs, Ipswich, MA, USA) for about 10 minutes. After two washing steps with DMEM (15 min), the cells were detached. Using fluorescence-activated cell sorting (FACS), single fluorescent cells were transferred into 96 well plates. After about 3 weeks, single cell clones were again stained using the SNAP-cell SiR dye and clonal cell lines with labeled mitochondria were selected. Expression of the COX8A-SNAP fusion protein was verified by Western blotting.

### Staining of live cells for STED nanoscopy

Cells were seeded in glass bottom dishes (ibidi GmbH, Martinsried, Germany) one day before the measurements. Cells were stained with DMEM containing 1 µM SNAP-Cell SiR (New England Biolabs) and optionally 0.1% (v/v) Quant-IT PicoGreen dsDNA reagent (Thermo Fisher Scientific, Waltham, MA, USA) (15 min, 37 °C). After removing the staining solution and two washing steps with DMEM, the cells were left in the incubator in DMEM for 15–30 minutes to remove unbound dye. Prior to imaging, DMEM was replaced with life cell imaging solution (Thermo Fisher Scientific).

### Staining of fixed cells for STED nanoscopy

Cells were seeded on coverslips and fixed with pre-warmed (37 °C) 8% formaldehyde in phosphate buffered saline for 7 min (PBS, 137 mM NaCl, 2.68 mM KCl and 10 mM Na_2_HPO_4_, pH 7.4). The cells were afterwards permeabilized with 0.5% Triton X-100 in PBS (5 min) and blocked with a solution of 5% (w/v) bovine serum albumin (BSA) in PBS (10 min, RT). Cells were labeled for ATP5I and double-stranded DNA with specific antisera (Proteintech, Rosemont, IL, USA, Abcam, Cambridge, UK). Primary antibodies were diluted in 5% BSA (w/v) in PBS and added to the samples (1 h, RT). Samples were washed several times with PBS and blocked with BSA solution (5 min, RT). The primary antibodies were detected with secondary anti-mouse antibodies labeled with Alexa Fluor 594 (Thermo Fisher Scientific, Waltham, MA, USA) or anti-rabbit antibodies (Jackson Immuno Research Laboratories, West Grove, PA, USA) custom labeled with the dye Abberior STAR RED (Abberior, Göttingen, Germany) (1 h, RT). The samples were washed five times with PBS and mounted in Mowiol mounting medium containing 0.1% (w/v) DABCO (Sigma Aldrich, St. Louis, MO, USA).

### STED nanoscopy

STED nanoscopy was performed using a quad scanning STED microscope (Abberior Instruments, Göttingen, Germany) equipped with a UPlanSApo 100x/1,40 Oil objective (Olympus, Tokyo, Japan). The pinhole was set to 0.7–1.0 Airy units. A pixel size of 20–25 nm was used.

For live STED imaging, SNAP-Cell SiR was excited at 640 nm and STED was performed at 775 nm wavelength. PicoGreen was excited at 485 nm wavelength. The fluorescence signal was detected using avalanche photo diodes with bandpass filters. For STED imaging of SNAP-Cell SiR, a gating of 0.75–8 ns was applied. Dwell times of 7–10 µs were used. For STED images, each line was scanned 4 to 8 times and the signal was accumulated. For the confocal images, each line was scanned once.

For dual color STED imaging of fixed cells, STAR RED was excited at 640 nm and STED was performed at 775 nm wavelength. AlexaFluor 594 was excited at 561 nm wavelength. The fluorescence signal was detected using avalanche photo diodes with bandpass filters and a gating of 0.75–8 ns was applied. Dwell times of 10 µs were used. Each line was scanned 3 times and the signal was accumulated.

### Image processing

No deconvolution was used. All main still images (Figs 1a,b and 2b) display unprocessed raw data without background subtraction. For time lapse recordings (Fig. 2c; Supplementary Movies S1–S4), photobleaching was compensated using the Bleach Correction function (histogram matching) in the Fiji software. For dual-color live-cell recordings, background subtraction was performed (5%-10% of the maximum signal).

### Transmission EM of HeLa cells

HeLa cells were grown on aclar discs (Plano, Wetzlar, Germany) to a confluency of about 60% and fixed with pre-warmed 2.5% glutaraldehyde in 0.1 M cacodylate buffer (pH 7.4, 1 h, RT). Samples were stored in the fixative over night to complete fixation (4 °C). Cells were washed three times with 0.1 M cacodylate buffer and were stained in 1% (w/v) osmium tetroxide in 0.1 M cacodylate buffer (pH 7.4, 1 h, RT). The samples were washed with distilled water (three times, each for 5 min) and stained en-bloc for 30 min in aqueous 1% (w/v) uranyl acetate (RT, in the dark). Dehydration was performed using an ethanol series of 30, 50, 70 and 100% (three times, each for 5 min) with a final dehydration step in propylene oxide (5 min). Cells were embedded in Agar 100 epoxy resin. Sections of 70 nm thickness were recorded on a Philips CM120 transmission electron microscope with a TVIPS 2k × 2k slow-scan CCD camera.

## Cell line availability

The COX8A-SNAP-tag cell line is available from the corresponding author for non-commercial use upon reasonable request.

## Supplementary information

Supplementary Movie S1

Supplementary Movie S2

Supplementary Movie S3

Supplementary Movie S4

Supplementary Information

## Data Availability

All raw data used to create the figures and videos in this paper are available from the corresponding author upon reasonable request.

## References

[1] Bereiter-Hahn J, Voth M (1994). Dynamics of mitochondria in living cells: shape changes, dislocations, fusion, and fission of mitochondria. Micros Resarch & Tech.

[2] Friedman JR, Nunnari J (2014). Mitochondrial form and function. Nature.

[3] Scheffler, I. E. Mitochondria, second edition. John Wiley & Sons, Inc. Hoboken, New Jersey, USA (2008).

[4] Cogliati S, Enriquez JA, Scorrano L (2016). Mitochondrial Cristae: Where Beauty Meets Functionality. Trends Biochem Sci.

[5] Jakobs S, Wurm CA (2014). Super-resolution microscopy of mitochondria. Curr Opin Chem Biol.

[6] Ji N, Shroff H, Zhong H, Betzig E (2008). Advances in the speed and resolution of light microscopy. Curr Opin Neurobiol.

[7] Sahl SJ, Hell SW, Jakobs S (2017). Fluorescence nanoscopy in cell biology. Nat Rev Mol Cell Biol.

[8] Sigal YM, Zhou R, Zhuang X (2018). Visualizing and discovering cellular structures with super-resolution microscopy. Science.

[9] Schmidt R (2009). Mitochondrial Cristae Revealed with Focused Light. Nano Letters.

[10] Shao L, Kner P, Rego EH, Gustafsson MG (2011). Super-resolution 3D microscopy of live whole cells using structured illumination. Nat Methods.

[11] Fiolka R, Shao L, Rego EH, Davidson MW, Gustafsson MG (2012). Time-lapse two-color 3D imaging of live cells with doubled resolution using structured illumination. Proc Natl Acad Sci USA.

[12] Huang X (2018). Fast, long-term, super-resolution imaging with Hessian structured illumination microscopy. Nat Biotechnol.

[13] Wäldchen S, Lehmann J, Klein T, van de Linde S, Sauer M (2015). Light-induced cell damage in live-cell super-resolution microscopy. Sci Rep.

[14] Kilian N (2018). Assessing photodamage in live-cell STED microscopy. Nat Methods.

[15] Keppler A (2003). A general method for the covalent labeling of fusion proteins with small molecules *in vivo*. Nat Biotechnol..

[16] Hinner MJ, Johnsson K (2010). How to obtain labeled proteins and what to do with them. Curr Opin Biotechnol.

[17] Gilkerson RW, Selker JML, Capaldi RA (2003). The cristal membrane of mitochondria is the principal site of oxidative phosphorylation. FEBS Lett.

[18] Stoldt S (2018). Spatial orchestration of mitochondrial translation and OXPHOS complex assembly. Nat Cell Biol.

[19] Liu X, Yang L, Long Q, Weaver D, Hajnoczky G (2017). Choosing proper fluorescent dyes, proteins, and imaging techniques to study mitochondrial dynamics in mammalian cells. Biophys Rep.

[20] Hockemeyer D (2009). Efficient targeting of expressed and silent genes in human ESCs and iPSCs using zinc-finger nucleases. Nat Biotechnol.

[21] DeKelver RC (2010). Functional genomics, proteomics, and regulatory DNA analysis in isogenic settings using zinc finger nuclease-driven transgenesis into a safe harbor locus in the human genome. Genome Res.

[22] Lukinavicius G (2013). A near-infrared fluorophore for live-cell super-resolution microscopy of cellular proteins. Nat Chem.

[23] Daum B, Walter A, Horst A, Osiewacz HD, Kuhlbrandt W (2013). Age-dependent dissociation of ATP synthase dimers and loss of inner-membrane cristae in mitochondria. Proc Natl Acad Sci USA.

[24] Brandt, T. *et al*. Changes of mitochondrial ultrastructure and function during ageing in mice and Drosophila. *Elife* **6**, 10.7554/eLife.24662 (2017). 10.7554/eLife.24662 PMC5580880 28699890

[25] McArthur Kate, Whitehead Lachlan W., Heddleston John M., Li Lucy, Padman Benjamin S., Oorschot Viola, Geoghegan Niall D., Chappaz Stephane, Davidson Sophia, San Chin Hui, Lane Rachael M., Dramicanin Marija, Saunders Tahnee L., Sugiana Canny, Lessene Romina, Osellame Laura D., Chew Teng-Leong, Dewson Grant, Lazarou Michael, Ramm Georg, Lessene Guillaume, Ryan Michael T., Rogers Kelly L., van Delft Mark F., Kile Benjamin T. (2018). BAK/BAX macropores facilitate mitochondrial herniation and mtDNA efflux during apoptosis. Science.

[26] Zick M, Rabl R, Reichert AS (2009). Cristae formation-linking ultrastructure and function of mitochondria. Biochim Biophys Acta.

[27] Harner, M. E. *et al*. An evidence based hypothesis on the existence of two pathways of mitochondrial crista formation. *Elife* **5**, 10.7554/eLife.18853 (2016). 10.7554/eLife.18853 PMC5138035 27849155

[28] Blum TB, Hahn A, Meier T, Davies KM, Kuhlbrandt W (2019). Dimers of mitochondrial ATP synthase induce membrane curvature and self-assemble into rows. Proc Natl Acad Sci USA.

[29] Kukat C (2015). Cross-strand binding of TFAM to a single mtDNA molecule forms the mitochondrial nucleoid. Proc Natl Acad Sci USA.

[30] Kopek BG, Shtengel G, Xu CS, Clayton DA, Hess HF (2012). Correlative 3D superresolution fluorescence and electron microscopy reveal the relationship of mitochondrial nucleoids to membranes. Proc Natl Acad Sci USA.

[31] Stoldt S (2019). Mic60 exhibits a coordinated clustered distribution along and across yeast and mammalian mitochondria. Proc Natl Acad Sci USA.

[32] Grotjohann T (2011). Diffraction-unlimited all-optical imaging and writing with a photochromic GFP. Nature.

[33] Brakemann T (2011). A reversibly photoswitchable GFP-like protein with fluorescence excitation decoupled from switching. Nat Biotechnol.

[34] Li D., Shao L., Chen B.-C., Zhang X., Zhang M., Moses B., Milkie D. E., Beach J. R., Hammer J. A., Pasham M., Kirchhausen T., Baird M. A., Davidson M. W., Xu P., Betzig E. (2015). Extended-resolution structured illumination imaging of endocytic and cytoskeletal dynamics. Science.

[35] Wang Chenguang, Taki Masayasu, Sato Yoshikatsu, Tamura Yasushi, Yaginuma Hideyuki, Okada Yasushi, Yamaguchi Shigehiro (2019). A photostable fluorescent marker for the superresolution live imaging of the dynamic structure of the mitochondrial cristae. Proceedings of the National Academy of Sciences.

