# Supplementary figures and images for: Live-cell STED nanoscopy of mitochondrial cristae
Source: Sci Rep. 2019 Aug 27;9:12419. doi: 10.1038/s41598-019-48838-2 (PMC6712041; doi:10.1038/s41598-019-48838-2)

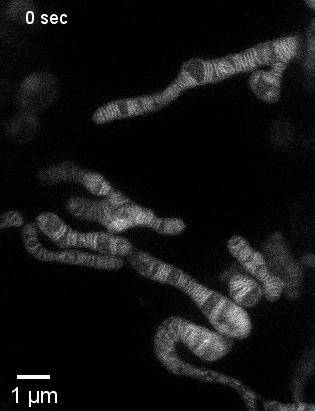

Supplement: Supplementary file 1 — Supplementary Movie S1 [file 41598_2019_48838_MOESM1_ESM.gif]

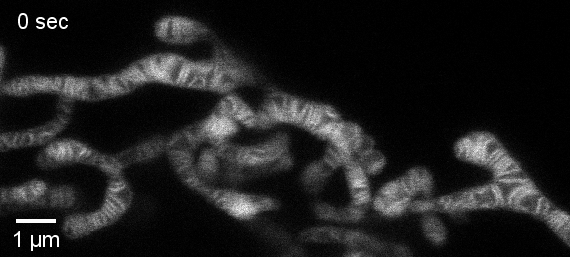

Supplement: Supplementary file 2 — Supplementary Movie S2 [file 41598_2019_48838_MOESM2_ESM.gif]

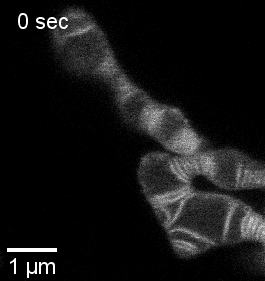

Supplement: Supplementary file 3 — Supplementary Movie S3 [file 41598_2019_48838_MOESM3_ESM.gif]

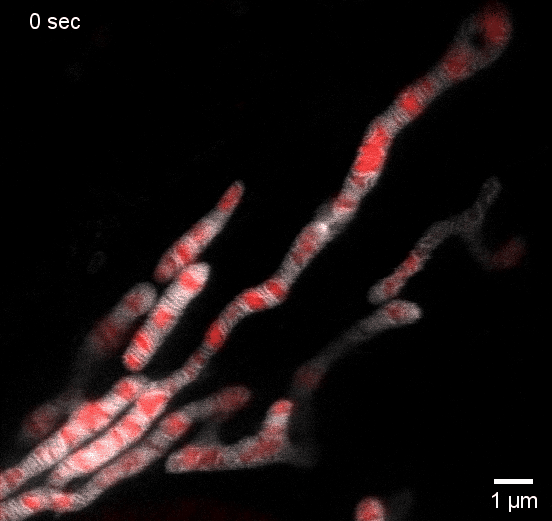

Supplement: Supplementary file 4 — Supplementary Movie S4 [file 41598_2019_48838_MOESM4_ESM.gif]
