# Supplementary material for: Live-cell STED nanoscopy of mitochondrial cristae
Source: Sci Rep. 2019 Aug 27;9:12419. doi: 10.1038/s41598-019-48838-2 (PMC6712041; doi:10.1038/s41598-019-48838-2)
Supplement: Supplementary file 5 — Supplementary Information [file 41598_2019_48838_MOESM5_ESM.pdf]

Supplementary Information for

Live-cell STED nanoscopy of mitochondrial cristae

Till Stephan[a,c], Axel Roesch[a,c], Dietmar Riedel[b], Stefan Jakobs[a,c]

[a]Department of NanoBiophotonics, Max Planck Institute for Biophysical Chemistry, 37077 Göttingen, Germany; [b]Laboratory of Electron Microscopy, Max Planck Institute for Biophysical Chemistry, 37077 Göttingen, Germany; [c]Clinic of Neurology, University Medical Center Göttingen, 37075 Göttingen, Germany

Correspondence to SJ (sjakobs@gwdg.de)

This PDF file includes:

Supplementary Figures S1-S3
Supplementary Figure Legends
Captions for Supplementary Movies S1-S4

Other supplementary materials for this manuscript include the following:

Supplementary Movies S1-S4

Supplementary Figure S1

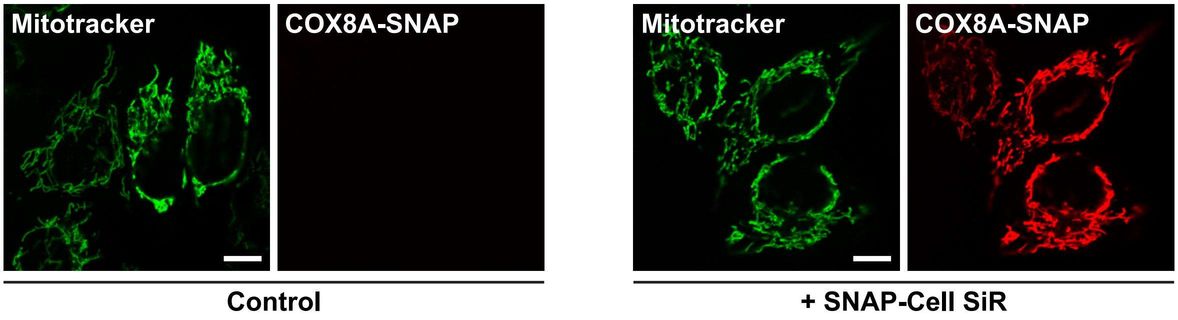

Supplementary Figure S1

Specificity of SNAP-labeling. HeLa cells stably expressing COX8A-SNAP fusion proteins were imaged without (Control, left) and after addition of the SNAP-Cell SiR dye to the growth medium (right). The mitochondria were highlighted with the mitochondria-specific dye Mitotracker Green. Scale bar: 10 µm.

Supplementary Figure S2

a

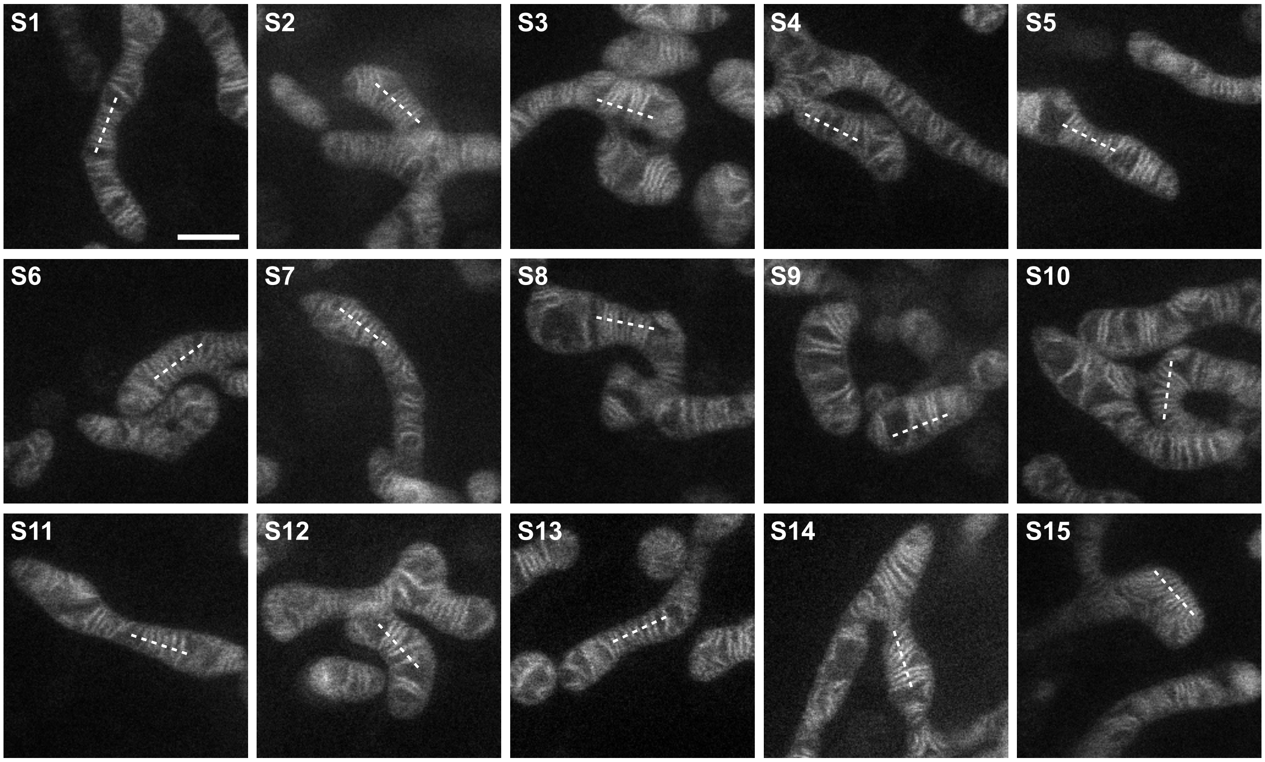

b

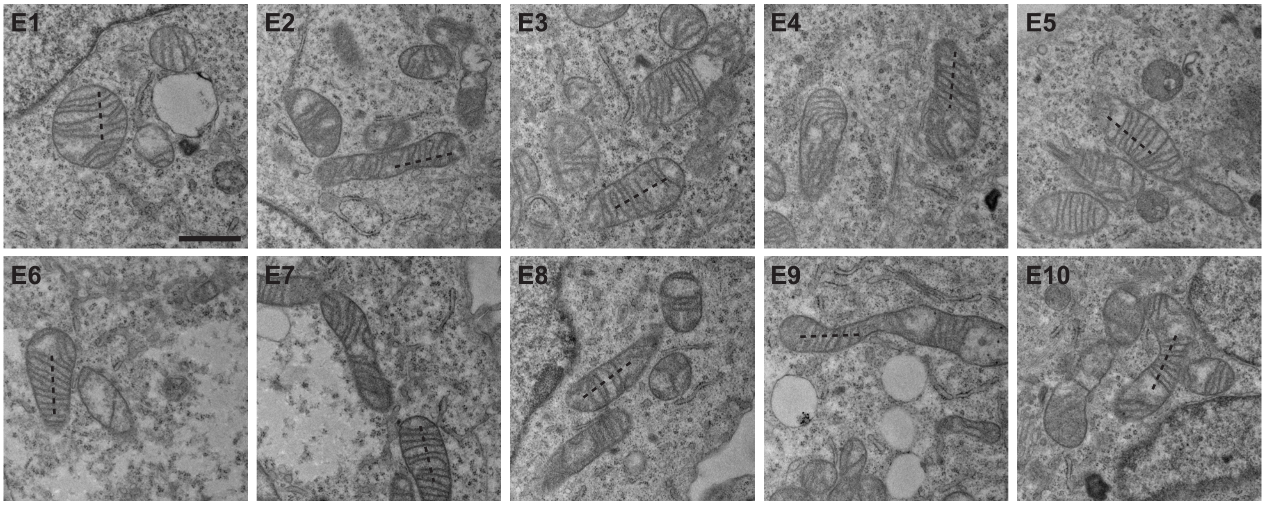

c

STED image	mean crista to crista distance in nm		SD	EM image	mean crista to crista distance in nm		SD
**S1**	100	±	22	**E1**	139	±	18
**S2**	111	±	60	**E2**	108	±	21
**S3**	155	±	72	**E3**	127	±	40
**S4**	156	±	71	**E4**	118	±	18
**S5**	113	±	28	**E5**	113	±	23
**S6**	134	±	49	**E6**	118	±	20
**S7**	110	±	32	**E7**	110	±	26
**S8**	100	±	20	**E8**	152	±	33
**S9**	147	±	70	**E9**	132	±	32
**S10**	107	±	16	**E10**	87	±	17
**S11**	124	±	38				
**S12**	140	±	59				
**S13**	134	±	50				
**S14**	123	±	20				
**S15**	88	±	17				
**mean**	**123 nm**			**mean**	**120 nm**		

Supplementary Figure S2

Quantification of crista-to-crista distances in mitochondria. a. Determination of crista-to-crista distances on live-cell STED images. HeLa cells stably expressing COX8A-SNAP were recorded by STED nanoscopy. On 15 manually selected mitochondria from nine different cells the fluorescence intensity was measured by line profiles (indicated by dashed lines, each line profile is 1 μm long). The crista-to-crista distances within the respective crista groups were estimated based on these measurements. **b.** Determination of crista-to-crista distances on transmission electron microscopy images. On manually selected mitochondria the crista-to-crista distances were estimated by line profiles (indicated by dashed lines, each line profile is 1 μm long). Electron micrographs were randomly selected from a total number of 93 images randomly taken from at least 10 different cells. **c.** Table of mean crista-to-crista distances on mitochondria determined on STED nanoscopy and EM images. The table shows the mean crista-to-crista distance for every line profile shown in (a) and (b). SD: Standard deviation. Scale bars: 1 μm.

Supplementary Figure S3

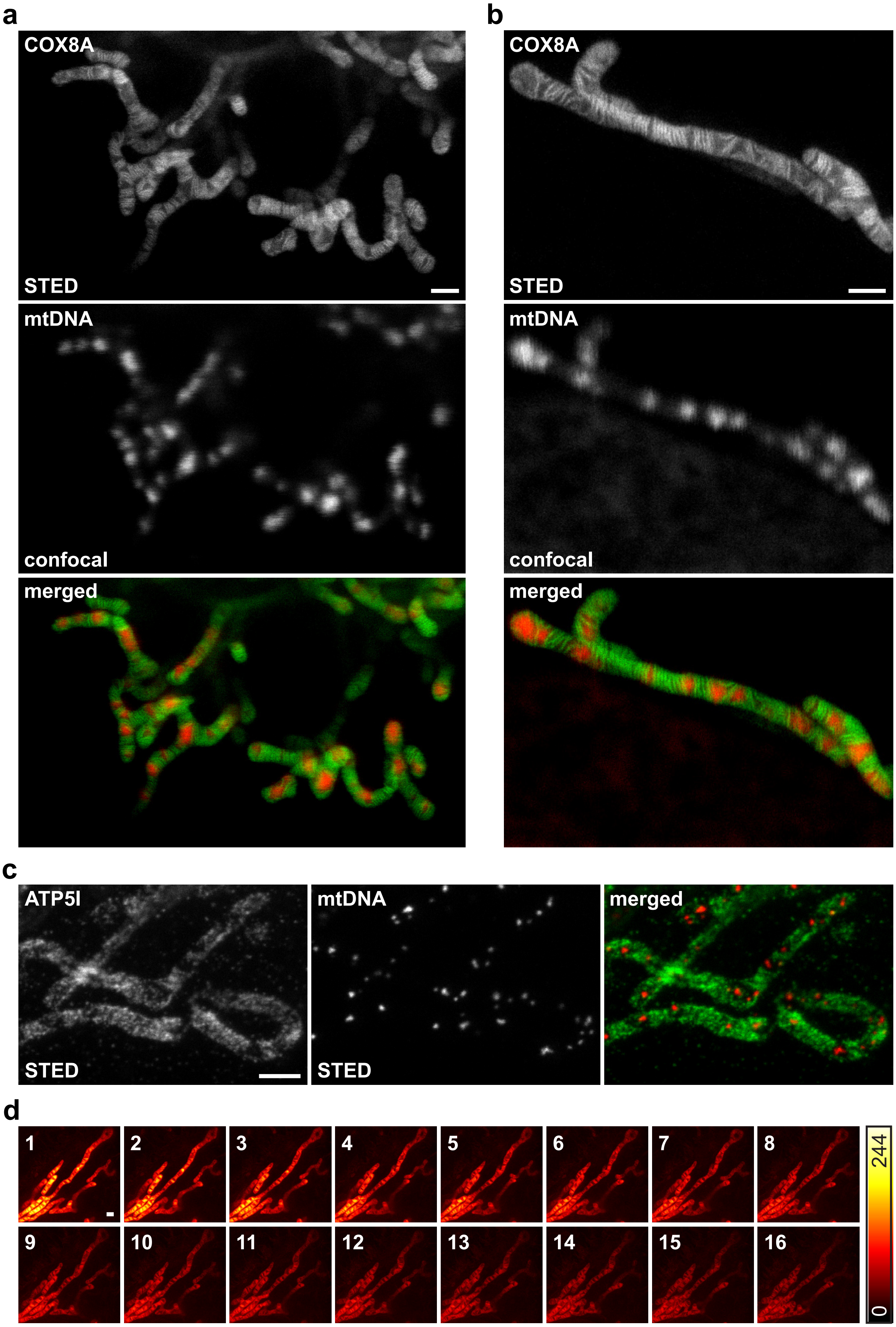

Supplementary Figure S3

STED nanoscopy. a, b. Live-cell dual-color recordings of the mitochondrial inner membrane and mtDNA. HeLa cells stably expressing COX8A-SNAP were labeled with SNAP-Cell SiR to highlight the mitochondrial inner membrane and with PicoGreen to label the mtDNA. SNAP-Cell SiR was recorded by STED nanoscopy and PicoGreen in the confocal mode. Shown are representative images from two different cells. **c.** STED nanoscopy of mtDNA and ATP5I in chemically fixed HeLa cells. HeLa cells were immunolabeled with antisera specific for double stranded DNA and against ATP5I, a subunit of the F_1F_o-ATP synthase. Cells were recorded with dual-color STED nanoscopy. **d.** Photobleaching in live-cell STED nanoscopy. HeLa cells stably expressing COX8A-SNAP were labeled with SNAP-Cell SiR and recorded by STED nanoscopy. Shown are the raw data images from an image series of 16 consecutive live-cell STED recordings. Recording time for one frame: 15 seconds. The same color coding is used for all images to demonstrate the amount of photobleaching. The same image data are shown in Supplementary Movie S4, but with an adjustment of the color coding to correct for photobleaching. In a-c, but not in d, we performed a background subtraction (5%-10% of the maximum signal). Scale bars: 1 µm.

Supplementary Movie S1 to S3

Live-cell time-lapse STED nanoscopy of mitochondria. HeLa cells stably expressing COX8A-SNAP fusion proteins were labeled using SNAP-Cell SiR and visualized with STED nanoscopy. Mitochondria were recorded every 15 (Movie S1), 10 (Movie S2) or 5 (Movie S3) seconds. Scale bar: 1 µm. The movies show raw data; photobleaching was compensated by adapting the color table. Scale bar: 1 µm.

Supplementary Movie S4

Dual-color live-cell microscopy of mitochondria. Hela cells stably expressing COX8A-SNAP fusion proteins were labeled with SNAP-Cell SiR and PicoGreen (to visualize mtDNA). Mitochondria were recorded every 15 seconds. COX8A-SNAP (grey) was recorded in STED mode, PicoGreen (red) in the confocal mode. Photobleaching was compensated by adapting the color table; to remove background, we subtracted 5 % of the maximal signal. Scale bar: 1 µm.
